# Correction: Highly potent and selective phosphatidylinositol 4-kinase IIIβ inhibitors as broad-spectrum anti-rhinoviral agents

**DOI:** 10.1039/d4md90022g

**Published:** 2024-06-12

**Authors:** Avinash G. Vishakantegowda, Dasom Hwang, Prashant Chakrasali, Eunhye Jung, Joo-Youn Lee, Jin Soo Shin, Young-Sik Jung

**Affiliations:** a Infectious Diseases Therapeutic Research Center, Korea Research Institute of Chemical Technology Daejeon 34114 Republic of Korea jsshin@krict.re.kr ysjung@krict.re.kr; b Department of Medicinal Chemistry and Pharmacology, University of Science and Technology Daejeon 34113 Republic of Korea; c Laboratory of Veterinary Virology, College of Veterinary Medicine, Chungbuk National University Cheongju 28644 Republic of Korea

## Abstract

Correction for ‘Highly potent and selective phosphatidylinositol 4-kinase IIIβ inhibitors as broad-spectrum anti-rhinoviral agents’ by Avinash G. Vishakantegowda *et al.*, *RSC Med. Chem.*, 2024, **15**, 704–719, https://doi.org/10.1039/D3MD00630A.

There were two typographical errors in Table 4 in rows 19 and 21 and a column alignment error for the PI4KIIIβ column. Table 4 should read as follows:

**Table:** Inhibitory effect of *N*-(4-methyl-5-arylthiazol)-2-amide derivatives (**1a–7f**)^a^ against PI4KIIIα and PI4KIIIβ

Compd.	R	IC_50_^b^ (μM)	
		PI4KIIIα	PI4KIIIβ
**1a**		>10	0.094 ± 0.006
**2**		>10	2.5 ± 0.46
**3**		>10	0.019 ± 0.001
**4**		>10	0.03 ± 0.003
**5a**	2-Cl	>10	0.16 ± 0.034
**5b**	3-Cl	>10	0.04 ± 0.003
**5c**	4-Cl	>10	0.16 ± 0.035
**5d**	3-F	>10	0.029 ± 0.006
**5e**	3-Me	>10	0.021 ± 0.005
**5f**	4-Me	>10	0.19 ± 0.073
**5g**	2-OMe	>10	0.06 ± 0.006
**5h**	3-OMe	>10	0.026 ± 0.004
**5i**	4-OMe	>10	0.16 ± 0.07
**5j**	3,5-(OMe)2	>10	0.0161 ± 0.0025
**5k**	3,4-(OMe)2	>10	0.072 ± 0.005
**5l**	3,4,5-(OMe)3	>10	0.066 ± 0.00
**6**		>10	0.014 ± 0.004
**7a**		>10	0.027 ± 0.006
**7b**		>10	1.9 ± 1.7
**7c**		>10	0.51 ± 0.531
**7d**		>10	0.027 ± 0.014
**7e**		>10	0.016 ± 0.005
**7f**		>10	0.0158 ± 0.0031
**Pleconaril**		>10	>10
**Rupintrivir**		>10	>10
**Enviroxime**		>10	0.064 ± 0.01

a All data were obtained from at least three independent experiments and the mean values of the standard deviations are listed.

b IC_50_: inhibitory concentration (μM) for 50% inhibition of each kinase enzyme.

The Royal Society of Chemistry apologises for these errors and any consequent inconvenience to authors and readers.

